# Internal Jugular Vein Fenestration and Duplication: Anatomical Findings, Prevalence, and Literature Review

**DOI:** 10.3389/fsurg.2020.593367

**Published:** 2020-11-13

**Authors:** Xiqian Wang, Liwei Peng, Haixing Guo, Juha Hernesniemi, Xuepeng Xiong, Hugo Andrade-Barazarte, Rongjun Qian

**Affiliations:** ^1^Department of Oral and Maxillofacial Surgery, Henan Provincial People's Hospital, Zhengzhou University, Henan University, Zhengzhou, China; ^2^Department of Neurosurgery, Henan Provincial People's Hospital, Zhengzhou University, Henan University, Zhengzhou, China; ^3^Department of Oral and Maxillofacial Head Neck Surgery, School & Hospital of Stomatology, Wuhan University, Wuhan, China

**Keywords:** internal jugular vein, fenestration, duplication, malformation, spinal accessory nerve, neck dissection

## Abstract

**Background:** Comprehensive knowledge of the internal jugular vein (IJV) regarding its anatomical variations and the pattern of its course is valuable for preventing unexpected injuries during surgical procedures or central venous access. IJV anatomical anomalies such as fenestration and duplication are rare, mainly represented by case reports, and intraoperative findings.

**Objective:** To present two additional cases of IJV anomalies and highlight its clinical presentation, anatomical characteristics, management, and prevalence through an extensive literature review.

**Methods and Case Reports:** From January 2017 to December 2018, we retrospectively collected data of 221 patients undergoing neck dissection (ND) procedures and identified two patients with IJV anomalies (fenestration and duplication) providing a clinical prevalence of ~0.9%. The IJV fenestration referred to an IJV bifurcation that reunites proximal to the subclavian vein, whereas in the IJV duplication both branches remain separated. In both of our cases, the spinal accessory nerve (SAN) crossed the window between the IJV branches.

**Conclusion:** Anatomical variations are more likely to be identified intraoperatively or incidentally, and due to the risk of SAN and vascular injury, special attention should be taken to identify them preoperatively in order to reduce the risk of iatrogenic injury and unexpected complications.

## Introduction

Neck dissection (ND) or cervical lymphadenectomy is a frequent procedure performed in the management of patients with head and neck cancer (1). Radical ND is associated with severe comorbidities and post-operative complications (2, 3). Therefore, during the last decades, to improve outcome and quality of life, special attention has been paid to reduce complications and comorbidities during ND procedures by preserving the internal jugular vein (IJV) and/or the spinal accessory nerve (SAN) (2–4). Based on that, successful surgical management of patients undergoing ND depends on recognizing patient-specific anatomic structures that may increase their risk of an adverse outcome.

The internal jugular vein (IJV) besides being the largest vessel in the neck and head, represents a relevant surgical anatomical landmark for adjacent structures such as: the carotid artery, vagus nerve, SAN and cervical lymph nodes (5). The IJV can present certain anomalies such as: duplication (referring to a bifurcation of the vein with each branch having a separate connection to the subclavian vein) or either fenestration (which refers to a bifurcation that reunites proximal to the subclavian vein) (6, 7). Therefore, identifying these anatomical variations is useful to avoid unexpected surgical complications or during central venous catheterization.

Anatomical anomalies (duplication or fenestration) of the IJV are commonly underreported and its prevalence is limited to few case reports (8–12). Therefore, we present two additional cases and highlight its clinical presentation, anatomical characteristics, management, and prevalence through an extensive literature review.

## Methods

### Literature Review

We performed a PubMed search from 1986 to 2020 for articles in English on anatomical variations of the IVJ with the following keyword(s): human AND (anatomical variations) AND (anomalies) AND (internal jugular vein) AND (duplication) AND (fenestration). Furthermore, we scrutinized the bibliographies of the retrieved articles for additional references (Table 1).

**Table 1:** Literature review.

**Number**	**Author**	**Year**	**Study cohort**	**Reported cases**	**IJV anomalies**	**Prevalence**
1	Prades et al. (13)	2002	750	3	Duplication	0.4%
2	Gardiner et al. (14)	2002	1	1	Bifurcation	–
3	Turan-Ozdemir et al. (15)	2004	1	1	Duplication	–
4	Alaani et al. (16)	2005	1	1	Duplication	–
5	Nayak et al. (17)	2006	1	1	Duplication	–
6	Downie et al. (6)	2007	1	2	Duplication	–
7	Biondi et al. (9)	2009	1	1	Fenestration	–
8	Ozturk et al. (18)	2010	1	1	Fenestration	–
9	Dogan et al. (19)	2010	1	1	Duplication	–
10	Wong et al. (20)	2010	1	1	Duplication	–
11	Thakur et al. (21)	2011	1	1	Fenestration	–
12	Kapre et al. (22)	2012	1	1	Duplication	–
13	Hashimoto et al. (7)	2012	123	4	Fenestration	3.3%
14	Bacchoo et al. (23)	2014	1	1	Duplication	–
15	Moreno-Sánchez et al. (12)	2015	1	1	Fenestration	–
16	Pegot et al. (24)	2015	1	1	Fenestration	
17	Deepak et al. (25)	2015	3	3	Fenestration, Duplication, Posterior tributary	–
18	Bathala et al. (8)	2015	1	1	Duplication	–
19	Cvetko (10)	2015	1	1	Fenestration	–
20	Contrera et al. (26)	2016	295	3	Fenestration, Duplication, Bifurcation	1%
21	Ibrahim et al. (11)	2016	2	2	Fenestration	–
22	Nayak et al. (27)	2017	1	1	Bifurcation	–
23	Cvetko et al. (28)	2017	1	1	Fenestration	–
24	Abakay et al. (29)	2019	1	1	Fenestration	
25	This manuscript	2020	221	2	Fenestration and Duplication	0.9%

### Study Cohort

From January 2017 to December 2018, we retrospectively reviewed a total of 221 patients who underwent ND due to oral malignancies, among them two patients showed IJV duplication or fenestration anomalies, with a clinical prevalence of ~0.9%.

## Clinical Cases

### Case 1

**Figure 1:**
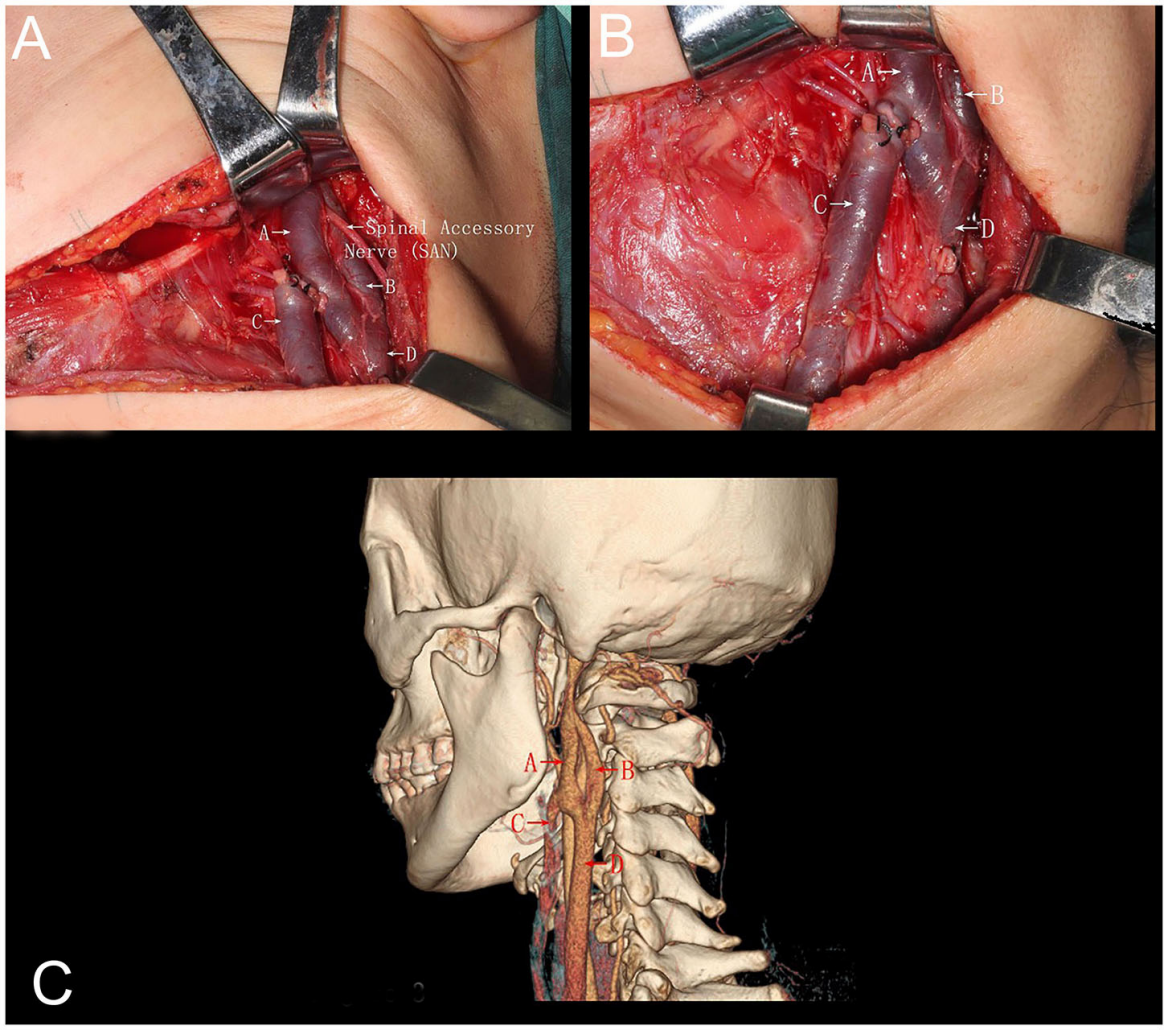
**(A)** Intraoperative picture demonstrating a left-side neck dissection. The internal jugular vein divided into two branches anterior (A) and posterior (B) from its origin at the jugular foramen. Both branches run downwards parallel to each other for ~4 cm in length and fused back together (D), demonstrating a window-like opening between the two branches. The anterior branch of the internal jugular vein received blood from an anterior tributary vein (C). Additionally, the spinal accessory nerve transversed this fenestration (A, anterior branch internal jugular vein; B, posterior branch internal jugular vein; C, anterior tributary vein; SAN, spinal accessory nerve; D, internal jugular fusion after fenestration). **(B)** Intraoperative picture of a left-side neck dissection, demonstrating the course of the anterior tributary vein (C) running down on the anterolateral region and deep into the platysma (A, anterior branch internal jugular vein; B, posterior branch internal jugular vein; C, anterior tributary vein; SAN, spinal accessory nerve; D, internal jugular fusion after fenestration. **(C)** Three-dimensional computed tomographic angiography corroborating the intraoperative findings (A, anterior branch internal jugular vein; B, posterior branch internal jugular vein; C, anterior tributary vein; SAN, spinal accessory nerve; D, internal jugular fusion after fenestration).

A 40-year-old woman presented with a lesion on the left margin of the tongue. Biopsy of this lesion showed a squamous cell carcinoma. The patient was clinically stage as T2N0M0 and underwent a left side partial resection of the tongue and suprascapulohyoid ND. Intraoperatively, the left IJV divided into two branches (anterior and posterior) after its cranial exit through the jugular foramen. This division continued distally for ~4 cm in length and both branches fused back together at the level of the central tendon of the digastric muscle (Figures 1A,B). Additionally, the SAN passed through the IJV fenestration. Before the fusion of the IJV branches, an anterior tributary was seen draining into the IJV from the anterolateral side of the platysma. The procedure went uneventful and without evidence of hemorrhages or SAN damage. Postoperative computed tomographic (CT) examination (Figure 1C) demonstrated the left IJV fenestration at the level of atlantoaxial intervertebral region in a retrospective view, suggesting that this anomaly could be clearly anticipated before performing a ND procedure.

### Case 2

**Figure 2:**
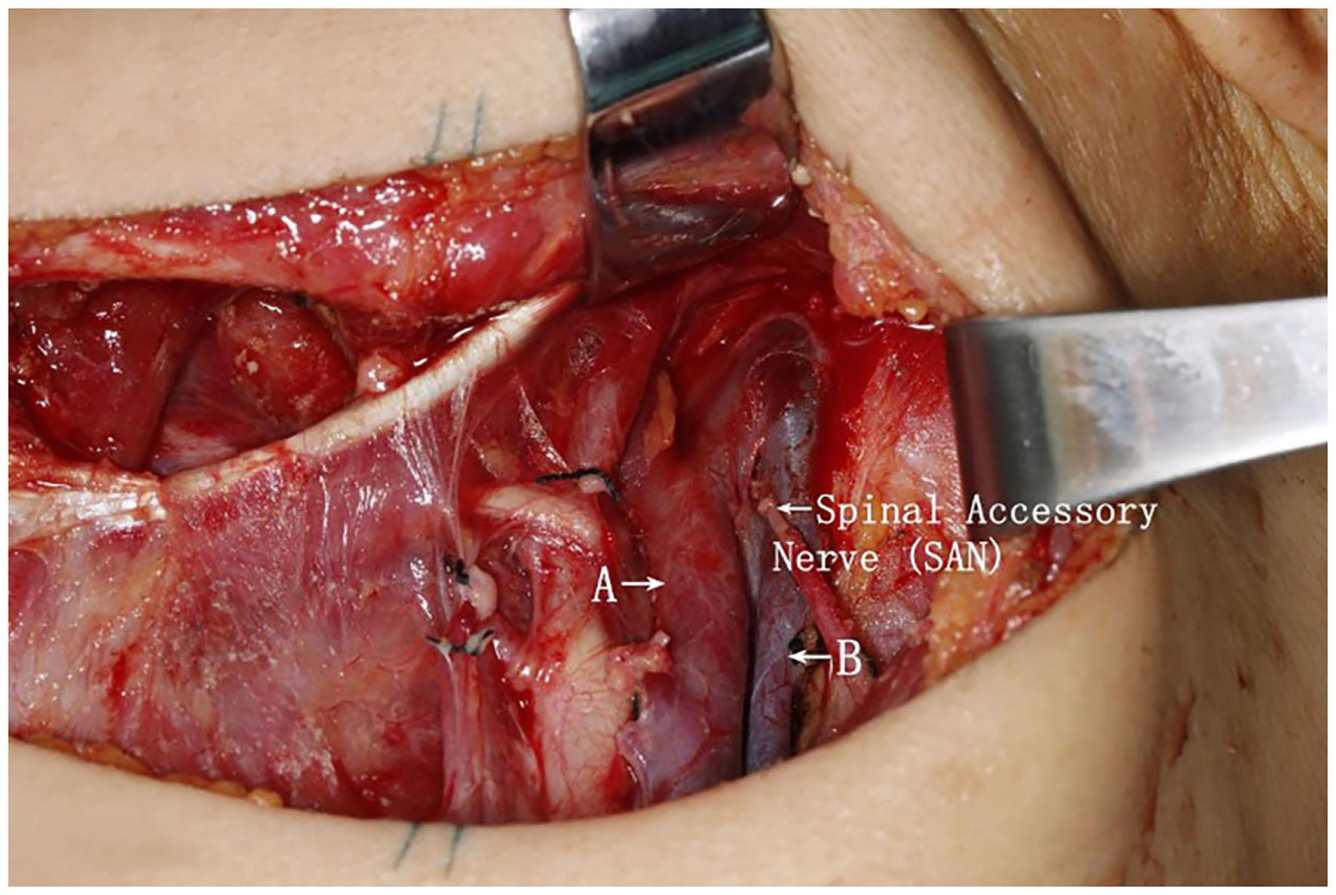
Intraoperative picture of a left-side neck dissection, demonstrating the division of the internal jugular vein into two branches, remaining unfused along the neck area until the level of the omohyoid muscle. This finding represented an internal jugular vein duplication. Additionally, the spinal accessory nerve bisected the two branches (A, anterior branch internal jugular vein; B, posterior branch internal jugular vein; SAN, spinal accessory nerve).

A 43-year-old woman who presented with a squamous cell carcinoma of the left mandibular gingiva. The patient was clinically staged as T2N0M0 and underwent a partial resection of the mandible and suprascapulohyoid ND. During the ND, the IJV divided into two branches (anterior and posterior) slightly above the mandibular angle and continued as two separate branches until the level of the omohyoid muscle. The spinal accessory nerve transversed the duplication from superficial to deep as it traveled toward the skull base (Figure 2). The perioperative course of the patient went uneventful and without evidence of hemorrhages or SAN damage.

## Discussion

The major vasculature of the head and neck region is represented by the carotid arteries and the jugular veins. These vascular structures are frequently accessed by head and neck surgeons during prophylactic or therapeutic cervical lymphadenectomy, or either by anesthetists or intensivists while placing central venous catheters for central venous pressure monitoring, drug administration, or volume resuscitation. Therefore, it is important to identify anatomical anomalies to prevent unexpected complications. Here, we present two additional cases of IJV anatomical anomalies (fenestration and duplication), and highlight its clinical presentation, anatomical characteristics, management, and prevalence through an extensive literature review.

### Prevalence

IJV duplications and fenestrations are rare, their prevalence has been calculated merely based on case reports and small case series (6–15, 18, 20–28, 30–33). Among them, Prades et al. (13) reported three cases of IJV (fenestration or duplication) malformations in 750 (French) ND cases, with a clinical prevalence of 0.4%. Moreover, Contrera et al. (26) demonstrated three cases of IJV fenestration among 295 cases of ND in the US, with a prevalence of 1% and Hashimoto et al. (7) identified four cases of IJV fenestration in 123 Japanese cases of ND, reporting a prevalence 3.33%. In our series of 221 Chinese patients undergoing ND procedures, the prevalence of IJV fenestration or duplication was 0.9%, which was in concordance with previously mentioned prevalence values.

### Identification and Clinical Implication

IJV anatomical anomalies (fenestration and duplication) can be observed preoperatively by computed tomographic angiography (CTA) or during contrast enhancement CT imaging as part of clinical and radiological staging of patients (7, 12, 26, 34). As demonstrated by Towbin and Kanal, who reported two cases of IJV fenestration identified through CTA (33). Additionally, a non-invasive imaging technique such as doppler ultrasound has been used to identify these variations, although it is not widely practiced (29, 35). However, despite the ability to identify these anomalies through contrast-enhanced CT imaging, it is only mentioned in seldom cases, thus reflecting the lack of attention paid to the venous system in the setting of a pathologic adenopathy.

Additionally, similar to previous case reports, our IJV fenestration and duplication were evinced intraoperatively by direct visualization, which still represents the most common diagnostic method reported in the literature (32).

Moreover, it is important to mention that our two cases showed no symptoms related to their IJV anomalies, in concordance with previous reports (7, 26, 32). On the contrary, only very rare cases of IJV are symptomatic, among them the majority of patients present with neck swelling, and very seldom dysphagia or dyspnea (15, 20, 36, 37). Therefore, this subset of patients might be misdiagnosed with laryngoceles or branchial cleft cysts (20).

It is worth to mention, that in our two cases no difference existed regarding diameter of the IJV branches (anterior and posterior branches), and that the common carotid artery was located behind the posterior branch. Even though this aspect has not been discussed, previous reports showed no asymmetry in diameter of the IJV duplications/fenestration (6, 7, 12, 20).

Furthermore, knowing the different types of IJV anomalies could be beneficial to avoid: (1) Iatrogenic injury of the IJV, if a duplication or a fenestration is not appreciated and the IJV is partially controlled during a ND, therefore causing copious bleeding, which might be challenging to control due to the presence of multiple feeders. (2) Risk of injury of the SAN, since this nerve often passes through the fenestration or duplication of the IJV and it is associated to quality of life and outcome (4).

### Ontogenesis

The etiology of IJV anomalies remains unclear. However, several hypotheses (vascular, neural, bony, and muscular) have been proposed in order to explain these anomalies (6, 26). In the vascular theory, the fenestration or duplication occurs due to inadequate condensation of the embryonic capillary plexus. Whereas, the neural theory suggests that branching of the IVJ is the result of obstructed growth by the SAN during development (which probably is consistent with cases of SAN bisecting the IJV). In the bony theory, aberrant ossifications or osteophytes could cause bony bridges responsible for venous partitioning. Finally, the muscular theory suggests the division of the IJV caused by the posterior belly of the omohyoid muscle (23).

## Conclusion

IJV fenestration and duplications are rare. These anatomical variations are more likely to be identified intraoperatively or incidentally, and due to the risk of SAN and vascular injury, special attention should be taken to identify them preoperatively in order to reduce the risk of iatrogenic injury and unexpected complications.

## Data Availability Statement

The original contributions presented in the study are included in the article/supplementary materials, further inquiries can be directed to the corresponding author/s.

## Ethics Statement

Due to the retrospective nature of the study informed consent was waived.

## Author Contributions

All authors contributed during the creation, data collection, preparation, editing, and revision of the following manuscript.

## Conflict of Interest

The authors declare that the research was conducted in the absence of any commercial or financial relationships that could be construed as a potential conflict of interest.
